# Potentially probiotic NPL 1334 strain of *Enterococcus durans* benefits rats with diet-induced hypercholesterolemia

**DOI:** 10.1186/s12896-024-00943-5

**Published:** 2025-01-17

**Authors:** Hannan Rashid, Haseeb Anwar, Fakhir Mehmood Baig, Imran Mukhtar, Tariq Muhammad, Arsalan Zaidi

**Affiliations:** 1 Pakistan Institute of Engineering and Applied Sciences (PIEAS), Nilore, Islamabad Pakistan; 2 National Probiotic Lab, National Institute for Biotechnology and Genetic Engineering-College (NIBGE-C) PIEAS, Faisalabad, Punjab 38000 Pakistan; 3 Department of Physiology, Government College University (GCUF), Faisalabad, Punjab, Pakistan

**Keywords:** Enterococcus, Hypercholesterolemia, Gut microbiota, Antioxidant potential, Probiotics

## Abstract

**Purpose:**

To study the potential of a candidate probiotic strain belonging to the *Enterococcus durans* species in alleviating hypercholesterolemia and improving the microbial milieu of rat gut.

**Methods:**

A previously isolated and characterized *E. durans* strain NPL 1334 was further screened in vitro for its bile salt hydrolyzation and cholesterol assimilation ability. An in vivo trial using diet-induced hypercholesterolemic rats was conducted to evaluate the effects of the administered test probiotic strain on the animal’s blood biochemical parameters such as total cholesterol (TC), high-density lipopolysaccharides (HDL), low-density lipopolysaccharides (LDL), triglycerides (TG), on body weight, oxidative stress markers, and its impact on intestinal and fecal microbiota as well as a histopathological examination of the test animal’s livers.

**Results:**

*E. durans* strain showed good bile salt hydrolyzing ability and ample cholesterol assimilation in vitro. Probiotic-fed hypercholesterolemic rats showed significantly lowered cholesterol, triglyceride and LDL levels. The body weight of probiotic-fed rats was reduced as compared to the control. *E. durans* also stimulated the growth of beneficial LAB in the intestine of experimental rats and did not harm the liver of the experimental rats.

**Conclusion:**

*E. durans* can be a natural therapeutic alternative to manage diet-induced hypercholesterolemia and may eventually enhance anti-cholesterolemic therapies.

## Introduction

Cholesterol is a biological molecule, necessary in the formation and function of cell membranes as well as vitamin and hormone synthesis in mammals. However, it is also involved in atherosclerosis, a degenerative disease where it blockages arteries [23] High levels of cholesterol in plasma promotes its deposition in the arteries as plaques causing the arterial lumen to become constricted, which is the hallmark of cardiovascular disease (CVD [85]). Elevated serum cholesterol adversely affects cardiovascular health, impacting many people globally [95], including Pakistan [99], where it causes many deaths [100]. In 2017, almost four million people were reported to have died from hypercholesterolemia, an alarming rise of 20% since the 1990s [38]. The World Health Organization ranks CVD as the leading cause of mortality until 2030, affecting 23.6 million people worldwide [93].

Fenofibrate is a peroxisome proliferator-activated receptor-α (PPAR- α) agonist used to treat hypercholesterolemia [78]. However, fenofibrate use is associated with some adverse outcomes [6]. Its long-term use adversely affects beta cell function and glucose-mediated insulin secretion [93], and it is ineffective in reducing CVD in type 2 diabetes mellitus patients [45]. To further compound this, the class II compounds defined by the Biopharmaceutical Classification Scheme (BCS), including Fenofibrate, are characteristically insoluble, making their therapeutic application difficult. They also have poor oral bioavailability (30% in human subjects), significantly curtailing their efficacy [40, 46]. It is, therefore, of utmost importance to find wholesome and natural cholesterol-reducing alternatives for treating CVD.

Probiotics present an affordable, noninvasive approach with little to no side effects in reducing CVD risk factors like total cholesterol (TC), low-density lipopolysaccharides (LDL), and other related inflammation markers [88]. Probiotics bring about reducing cholesterol in multiple ways, the most notable being bile salt hydrolase (BSH) activity, followed by cholesterol adhesion to the cell surface during bacterial growth [27, 81]. BSH-mediated bile salt deconjugation reduces the enterohepatic circulation of bile salts. Deconjugated bile salts are not absorbed as effectively as their conjugated counterparts because of their relative insolubility, resulting in their expulsion from the body via the feces [97]. This pulls more of the circulating cholesterol for synthesizing new bile acids *de novo*, eventually depressing serum cholesterol levels [25, 32, 56]. The hypocholesterolemic activity of probiotics is also attributed to their ability to thwart cholesterol micelle formation necessary for intestinal absorption [59] Furthermore, probiotics in rats can also bolster bile acid synthesis in the liver, lower plasma LDL-cholesterol levels by downregulating farnesoid X receptor- fibroblast growth factor 15 enterohepatic axis, and enhance liver LDL receptor expression [33].

Enterococci are a part of the human gut microbiome from infancy to old age. *Enterococcus* strains hold potential as cholesterol-lowering agents and bolstering immunity because of their robust BSH-mediated catalysis of bile acids [43, 53]. Minute changes in serum cholesterol brought on by probiotic administration significantly reduce the threat of coronary artery disease (CAD) [44].

So, to find an alternative natural therapy for cholesterol reduction, an *Enterococcus durans* strain previously isolated from the gut of a desi chicken breed and characterized for functional and safety attributes [73] was tested in vitro for BSH function and cholesterol assimilation. This was then followed by evaluating it in vivo against dietary-induced hypercholesterolemia in a rat model and monitoring its effect on biochemical parameters like TC, HDL, LDL, TG, alanine aminotransferase (ALT) and aspartate aminotransferase (AST), total antioxidant capacity (TAC) and total oxidative stress (TOS). Microbial characterization of rat gut and feces and histopathological examination of the liver were also performed.

## Materials and methods

### Bacterial cultures and growth conditions

*E. durans* was grown aerobically in MRS (deMan Rogosa & Sharpe) broth at 37^o^C. Stock cultures of *Enterococcus durans* in 25% (v/v) glycerol of desi chicken gut origin that we have reported earlier [72] were obtained from the culture collection of National Probiotic Lab (NPL), NIBGE for experimentation.

### Detection of bile salt hydrolysis activity

MRS agar plates augmented with 0.5% (wt/vol) one of the bile salts (tauro and glyco-conjugates of cholic, glycocholic and deoxycholic acid)) and CaCl_2_ (0.37 g/l) were used for assessing BSH activity. Wells were made by puncturing the agar surface, and 20 µl overnight culture of NPL 1334 was inoculated in plates. The plates were then anaerobically incubated at 37 ^o^C for 24 h. The inhibition zones surrounding the bacterial growth were measured (mm) by using a digital vernier caliper [92].

### Cholesterol assimilation

Cholesterol removal activity was performed using cholesterol PEG (Sigma-Aldrich, USA) according to a previously reported method [5]. Bacterial cultures were allowed to grow (48 h) in MRS broth medium supplemented with 100 µg/ml cholesterol PEG; following growth, the culture was centrifuged at 8000 x g for 5 min and supernatant (500 µL) was collected and mixed with absolute ethanol (1 ml) and 33% (w/v) KOH solution (500 µL) shaken and incubated at 37 ^o^ C for 15 min. After incubation, 1.5 ml of hexane and 1 ml of deionized water were added and kept at room temperature to separate the phases. The hexane layer was then shifted to a separate tube, and once dried, the reagent (50 mg/ml of o-phthalaldehyde in acetic acid) was added and carefully mixed. To this, concentrated H_2_SO_4_ (250 µl) was added, followed by 20 min incubation, and then absorbance was measured at 570 nm. Assimilated cholesterol was calculated using a standard curve comprising various cholesterol concentrations (0–100 µg/mL) plotted against the absorbance values.

### In vivo trial and analysis

#### Experimental layout

Thirty male albino rats aged about 8 weeks were obtained from the Animal Breeding Institute of Government College University, Faisalabad. Animals were placed in well-ventilated polypropylene cages (1 rat/cage). Room temperature was maintained at 22 ± 2 ^o^C, humidity at 50% ± 5%, and the cages were subjected to a daily 12/12 h light/dark cycle. Following a week of acclimatization, the animals were randomly split into five groups, with six animals per group. The groups were designated as control (C1), Hypercholesterolemic diet (HCD) fed (C2) and experimental groups (G1, G2 and G3). All test animals were ensured to have the same weight (25 g) irrespective of their group placement. Regular chow feed (Table 1) was fed to the control group (C1), while a high-fat diet comprising 3% lard, 12% corn oil, 0.5% cholesterol and 84.5% regular chow diet was provided to the other groups for 10 consecutive days to induce hyperlipidemia in the animal. After 10 days total cholesterol level was checked to ensure hypercholesterolemia is induced. Approximately 200 µl of a suspension of an overnight culture of the test probiotic strain corresponding to a concentration of 1 × 10^10^ CFU/ml, was provided daily via oral gavage for 28 days to G1. The animals in group G2 received Fenofibrate at a concentration of 20 mg/kg body weight; the test probiotic strain (200 µl of 1 × 10^10^ CFU/ml) and fenofibrate (10 mg/kg of body weight) were given in combination to G3. After completion of the experimental period, animals were euthanized by a single intraperitoneal anesthetic dosage of pentobarbital sodium (50 mg/kg). A record of the food intake and weight of animals was kept throughout the experiment.

**Table 1:** Percentage composition of normal chow diet

Ingredient	Percentage
Corn meal	52
Wheat flour	8
Soybean meal	27.5
Wheat bran	11
Methionine	0.1
Lysine	0.1
Vitamins	0.02
Sodium Chloride	0.2
CaCO_3_	1
Minerals	0.08

### Microbial recovery from animal gut

After 28 days, the rats were slaughtered following recommended ethical guidelines. Half a gram of intestinal and fecal material from each group (three samples from each) was used to analyze microbial contents. The material was suspended in 10 ml of standard saline solution (0.9% w/v NaCl), and tenfold dilutions were made, which were then plated on MRS agar to recover presumptive LAB isolates. Colony morphologies were noted after incubating the plates at 37º C for 24 h. The identity of the isolates collected from gut and fecal samples was molecularly confirmed using 16 S rDNA sequencing. Purified genomic DNA was obtained from cultures of a single colony grown in MRS broth using a commercial extraction kit (00821928/ Thermo Scientific), and the V3 –V4 regions of 16 S rRNA gene was amplified using specific primers 357 F (CCT ACG GGA GGC AGC AG) and 926R (CCG TCA ATT CMT TTR GT). Partial sequencing was commercially performed using an automated DNA analyzer (Macrogen, Korea). The identified sequences were deposited in the NCBI database, and their accession numbers were obtained.

### Assessing blood chemistry

Three rats randomly picked from the control and treated groups on the 11th and 28th days of the trial were used for recording blood biochemical parameters. Blood was collected from the tail vein in 1.1 ml clot activator tubes (cat no. 18040306, Hebei Xinle Sci & Tech Co. China) without additives and anticoagulants. Following blood coagulation, the serum was centrifugally partitioned from blood cells (2000 x g for 8 min), and the samples were freeze-dried and stored at -20 ^o^C for further use. Following the manufacturer’s instructions, the biochemical parameters (TC, HDL, LDL, TG, ALT, and AST) were checked using a clinical analyzer (Microlab-300, Merck, Germany).

### Antioxidative ability of test strain and animals

#### Assessing the free radical scavenging ability of test strain

An assay was performed to test the free radical scavenging potential of the supernatants of cultures of the test strains, according to the protocol described by [8]. The reaction reagent was made by mixing ABTS (7 mM) in water with potassium persulfate (2.45 mM) to produce radicals. The mixture was placed in the dark before use. The absorbance of ABTS stock solution diluted in methanol was set at 0.7 ± 0.030 at 730 nm, centrifugation was performed, and the supernatant obtained was further diluted with methanol (1:10) for inhibition with Trolox (0.5 mg/ml) used as a standard. The test supernatant (50 µl) was mixed with 250 µl of ABTS reagent, and absorbance was measured at 730 nm. The absorbance value is inversely proportional to scavenging activity. The following equation measured percentage inhibition:

$$\%\rm inhibition\, = \,1{\text{ - }} - {\text{As/Ac}} \times {\text{100}}$$

Where As is the absorbance of the sample and Ac is the absorbance of control.

The blood serum’s antioxidant capacity was examined based on 2,2 –azinobis 3-ethylbenzothiazoline-6-sulfonate (ABTS) color bleaching. Biochromatic wavelength (660 and 870 nm) was adjusted on the semi-autoanalyzer (BioSystem BTS 330, Spain), and vitamin C standards were calibrated at concentrations of 0.3, 0.6, 0.9, 1.2, and 1.5 mmol/l [65].

A colorimetric assay was used to determine total oxidative stress (TOS) in the serum samples, and Xylenol Orange dye was used in the reagent. A semi-auto analyzer (BioSystem BTS ^®^ 330, Spain) measured absorbance at wavelength 660 nm. The standard curve was calibrated using five standards of hydrogen peroxide (5, 10, 15, 20 and 25 µmol/l) [4].

### Malondialdehyde analysis (MDA)

The MDA level of rat liver was measured according to a previously described method [35] Liver tissue was homogenized (10% w/v) in chilled 0.15 M potassium chloride. Homogenate (1mL) was incubated in a metabolic shaker at 37 ^o^C for 2 h. One ml of (10% w/v) trichloroacetic acid was added in homogenate, and centrifugation was performed at 3000 g for 8 min. One milliliter of clear supernatant was mixed with the same amount of 0.67% (w/v) of 2-thiobarbituric acid and placed in hot water bath for 10 min. Samples were allowed to cool at room temperature and then diluted with distilled water (1 ml). Absorbance was measured at 530 nm, and MDA concentration was recorded using tetraethoxy-propane as standard.

### Histopathology of rat liver

On the 28th day of the study, the rats were euthanized via abdominal aorta exsanguination, and the liver was removed, weighed, and prepared for morphological examination. The livers from each group’s rat carcasses were sliced into portions of uniform size, three of which were then immediately placed in formalin (10% v/v) for further analysis. Standard procedures for histopathological analysis like fixation, dehydration, clearance, impregnation, blocking and mounting were performed [42], followed by staining with hematoxylin and eosin (H&E) and the slides were examined under a brightfield microscope (Olympus, BX63, Japan). The degree of hepatocyte damage was determined by a senior pathologist via a point scoring method using an ordinal scoring scale: point 0, no injury evidence; point 1, minimal injury with less fibrous content, moderate portal vein size and restoration of hepatocellular structure; point 2 modest to severe injury with hepatocyte death, high-fat accumulation in tissue, disruption of cellular content and portal vein deformation.

### Ethical statement

Animals for trial were housed in the animal care facility of the Department of Physiology, Government College University Faisalabad, following trial approval by the departmental biomedical ethical committee. The experimental design, procedures, housing, care and handling were done according to globally recommended procedures [13, 87].

### Statistical analysis

Info-Stat (FCA-UNC., Cordoba, AR) software was used for statistical analysis. Mean differences among experimental groups were determined by One-way analysis of variance (ANOVA) with treatment and block as fixed effects [28]. For heterogenous variances, data transformation was done to normalize. Anderson-Darling Test was used to evaluate the normality of the data set [62]. A randomized complete block design (RCBD) was used, and the rats were divided into five treatments, with three replicates per group and two rats per replicate. Tukey multiple comparison test in Graph Pad prism (version 6) was used to calculate statistical differences among means. A P-value of < 0.05 was regarded as statistically significant. All the experiments were done in triplicate unless indicated otherwise.

## Results

### Detection of BSH activity and cholesterol assimilation

The ability to hydrolyze primary and secondary bile salts was prominent in the *E. durans* strain NPL 1334. This strain could hydrolyze three of the four conjugated bile salts tested. The diameter of the halo zones of salt precipitation were recorded to be in the range of 10–13 mm. Cholesterol assimilation activity was profound in *E. durans* strain, which could assimilate up to 92% of the free cholesterol in broth media (data not shown).

### Probiotic performance in vivo

#### Microbiological analysis

**Fig. 1:**
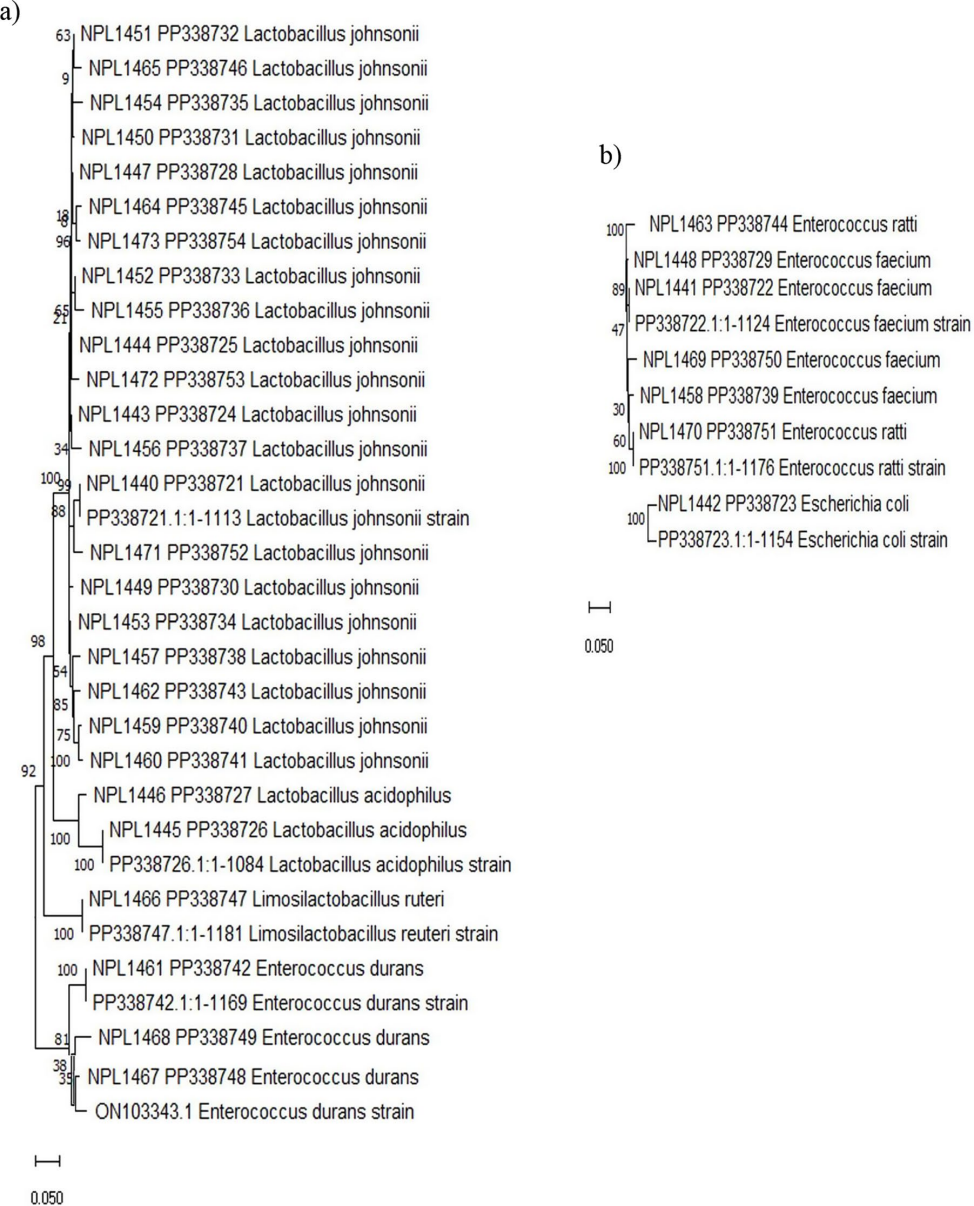
**a**). Phylogenetic relativeness of strains of various LAB spp. isolated from the gut and feces of rats administered the *E. durans* strain, **b**) Enterococcal strains recovered from the control and fenofibrate fed group rat’s gut and feces based on 16 S rDNA gene sequences. The tree was constructed with MEGA XI software using neighbor joining method

The microbiological analysis of the gut and feces of experimental rats was performed to explore the impact of *E. durans* and fenofibrate. There were substantial differences in the type of species collected from the intestines of animals subjected to various treatments. More colonies (an increase of ~ 60%) appeared on the MRS plates carrying the sample from G1 and G3 that received the test probiotic strain compared to C1, C2 and G2 (Table 2). The bacterial colonies isolated from the *E. durans* strain administrated group (G1 and G3) genetically matched to LAB spp. *Enterococcus durans* (11.11%), *Lactobacillus johnsonii* (77.77%), *Lactobacillus acidophilus* (7.40%) and *Limosilactobacillus reuteri* (3.7%), (Fig. 1a) whereas *Enterococcus faecium* (57.14%), *Enterococcus ratti* (28.57%) and *Escherichia coli* (14.28%) (Fig. 1b) were found in the control and Finofibrate administered group. The 16 S rRNA gene sequences were submitted to the NCBI database, and accession numbers PP338721-PP338754 were obtained (Fig. 1a & b).

**Table 2:** Effect of probiotics on the gut and feces of hyperlipidemic rats

Selective medium	Control 1	Control 2	Group 1^a^	Group 2^b^	Group 3^c^
MRS Agar	2.4 × 10^6*^	1.7 × 10^6^	7.1 × 10^8***^	5.3 × 10^6*^	6.8 × 10^7**^

ns, *,**,***,****Means with different superscripts in each row are significantly different with control group 2. Where ns = non-significant, *P < 0.05, **P < 0.01, ***P < 0.001

^a^Group 1: *Enterococcus durans*, ^b^Group 2: Fenofibrate, ^c^Group3: *Enterococcus durans* + Fenofibrate

#### Lipid profile and liver enzymes

**Fig. 2:**
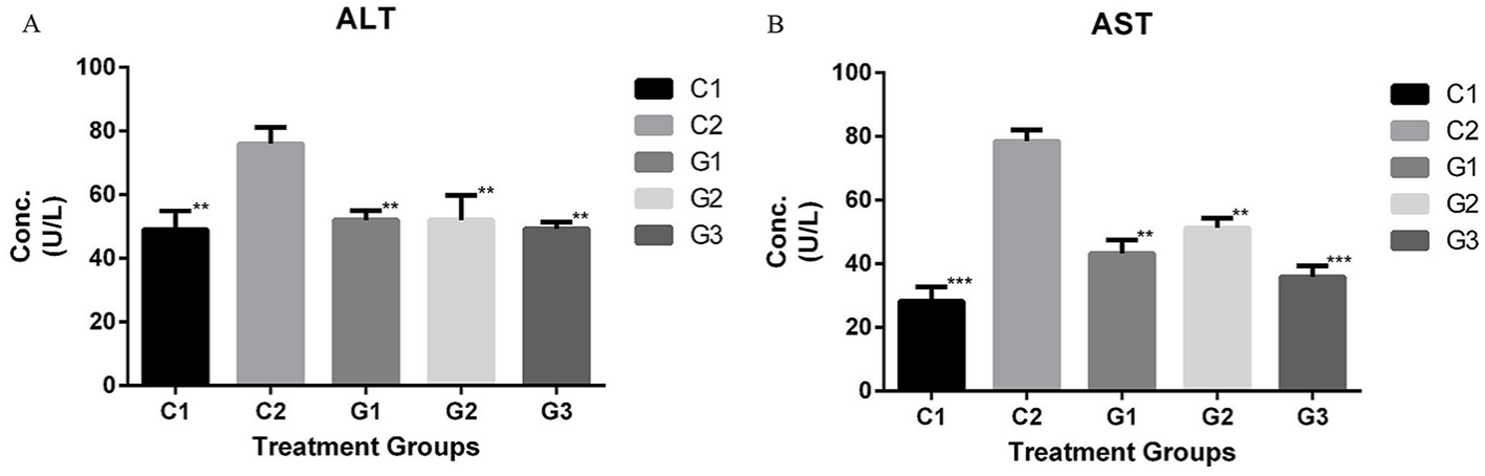
Comparison of liver enzymes **A**) ALT and **B**) AST of control and treated groups. Where, ^C1^Control, ^C2^HCD fed rats, ^G1^Probiotic Group, ^G2^Fenofibrate Group, ^G3^Probiotic + Fenofibrate Group. Where ns = non-significant, *P < 0.05, **P < 0.01, ***P < 0.001

The effect of probiotic and fenofibrate administration on the blood parameters of hyperlipidemic rats is shown in Table 3. Total cholesterol was significantly decreased in treatment groups G1, G2 and G3 compared to C2. Similarly, TG and LDL levels were significantly reduced in all treatment groups compared to control C2. A considerable increase in HDL level was seen in G1 and G3, whereas the HDL level of G2 was comparable with C2. The level of liver enzymes ALT and AST was significantly reduced in all treatment groups compared to C2 (Fig. 2).

**Table 3:** Biochemical parameters and body weight

Parameters	Control 1	Control 2	Group 1^a^	Group 2^b^	Group 3^c^
TC^1^ (mg/dL)	227.19 ± 26.21^****^	352.92 ± 5.49	271.86 ± 16.22^****^	244.95 ± 5.72^****^	190.91 ± 16.15^****^
TG^1^ (mg/dL)	149.66 ± 6.79^****^	205.00 ± 6.16	162.32 ± 3.93^****^	168.33 ± 3.39^****^	150.66 ± 3.85^****^
LDL^1^ (mg/dL)	172.67 ± 22.90^****^	309.48 ± 7.95	207.96 ± 28.91^****^	211.86 ± 28.09^****^	172.23 ± 22.47^****^
HDL^1^ (mg/dL)	63.92 ± 1.58^****^	24.90 ± 3.36	46.15 ± 3.08^*^	38.95 ± 2.65^ns^	54.23 ± 3.55^**^
Body weight (g)	155 ± 6.01^****^	263 ± 4.89	210 ± 4.54^****^	215 ± 8.73^****^	201 ± 3.29^****^
Liver MDA (pmol/mg protein)	9.32 ± 0.9^ns^	11.9 ± 0.5	6.22 ± 0.6^**^	8.47 ± 0.4^*^	5.14 ± 0.9^***^

ns, *,**,***,****Means with different superscripts in each row are significantly different with control group 2. Where ns = non-significant, *P < 0.05, **P < 0.01, ***P < 0.001, ****P < 0.0001

^1^Result of biochemical parameters tested from blood serum

^a^Group 1: *Enterococcus durans*, ^b^Group 2: Fenofibrate, ^c^Group3: *Enterococcus durans* + Fenofibrate

#### Animal body weight

The body weight of the control and treatment group rats was recorded on the 1st and 28th day of the experiment. Body weight at the start of the experiment was comparable in all groups; however, it was significantly reduced in all groups (G1, G2 and G3) at the end of the experiment when compared with C2 (Table 3).

#### Antioxidant capacity of the test strain

The cell-free supernatant of *E. durans* strain NPL 1334 was screened for ABTS radical scavenging. The antioxidant ability of the test strain was a quarter less (66.38%) than that of the standard Trolox (88.64%).

**Fig. 3:**
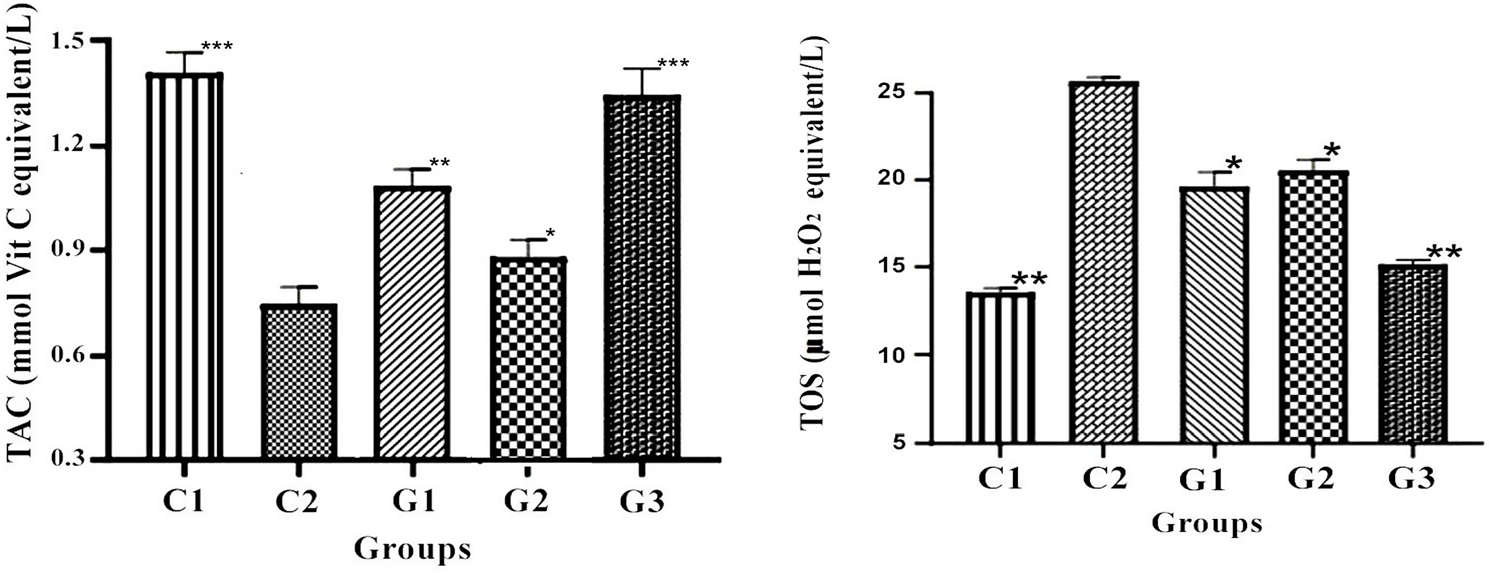
Total oxidant status (TOS) and Total Antioxidant Capacity (TAC) of control and treated groups. Where, ^C1^Control, ^C2^HCD fed rats, ^G1^Probiotic Group, ^G2^Fenofibrate Group, ^G3^Probiotic + Fenofibrate Group. Where ns = non-significant, *P < 0.05, **P < 0.01, ***P < 0.001

The current investigation demonstrated that probiotic supplementation decreased oxidative stress by generating more antioxidants in treatment groups than control C2, as illustrated in Fig. 3. The effect of feeding the test probiotic and fenofibrate on MDA levels of HFD-fed rats is shown in Table 2. The liver MDA level in the HFD diet group was significantly higher but equivalent to the fenofibrate-administered group. However, levels were dramatically lowered in test probiotic-treated groups compared to the HFD-fed group.

#### Gross examination of rat liver

**Fig. 4:**
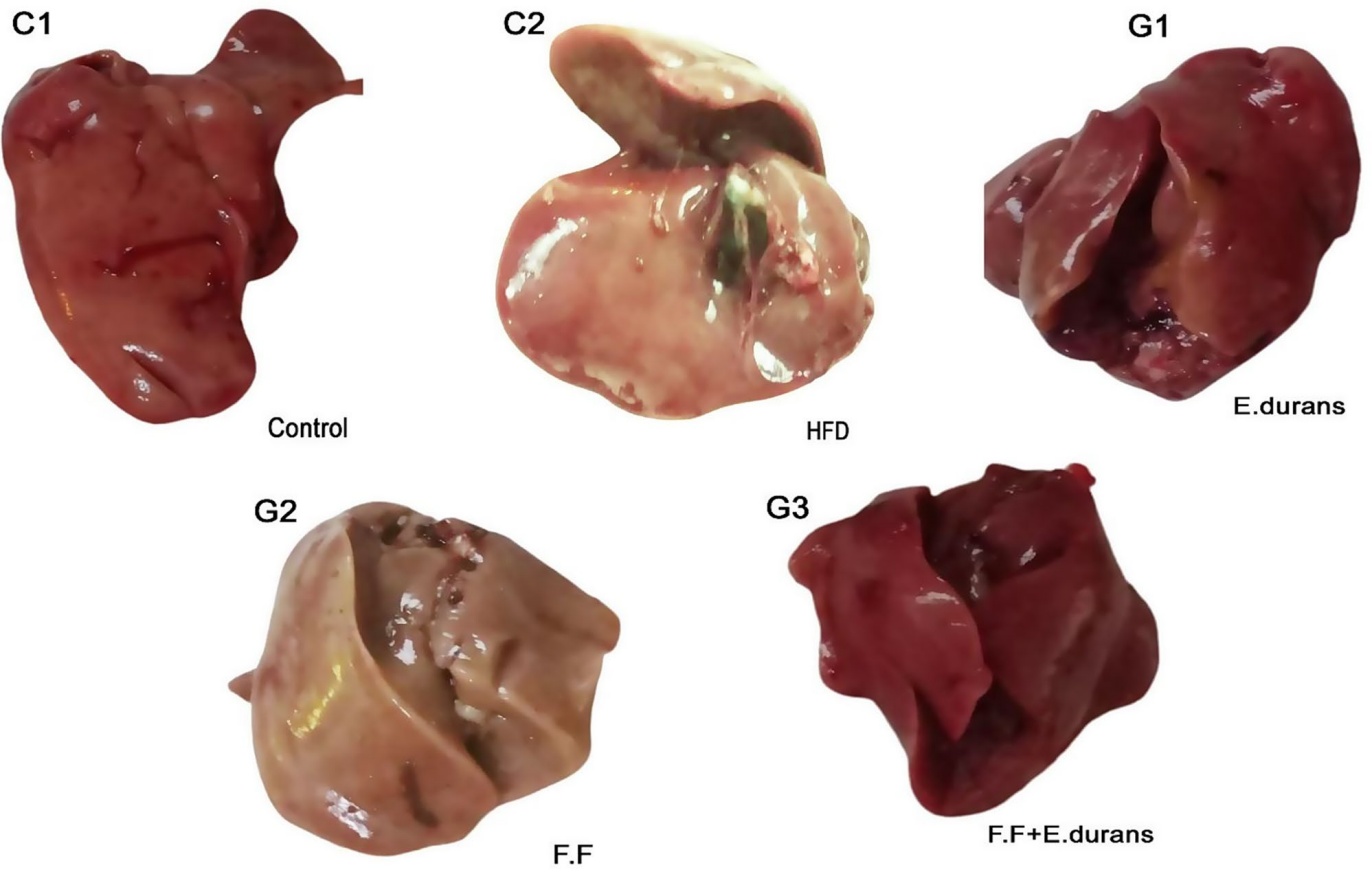
Gross examination of control and experimental group’s rat liver. Where, ^C1^Control, ^C2^HCD fed rats, ^G1^Probiotic Group, ^G2^Fenofibrate Group, ^G3^Probiotic + Fenofibrate Group

The results have shown that high-fat diet feeding resulted in significantly greasy and pale-looking livers in groups C2 and G2 groups (Fig. 4) in comparison to C1. Livers from animals in groups C1, G1, and G3 showed regular appearances compared to those in C2 and G2. The liver color was adjudged most optimal in the probiotic-administered group G1 among all the experimental groups, including the control group C1.

#### Histopathology of rat liver

**Fig. 5:**
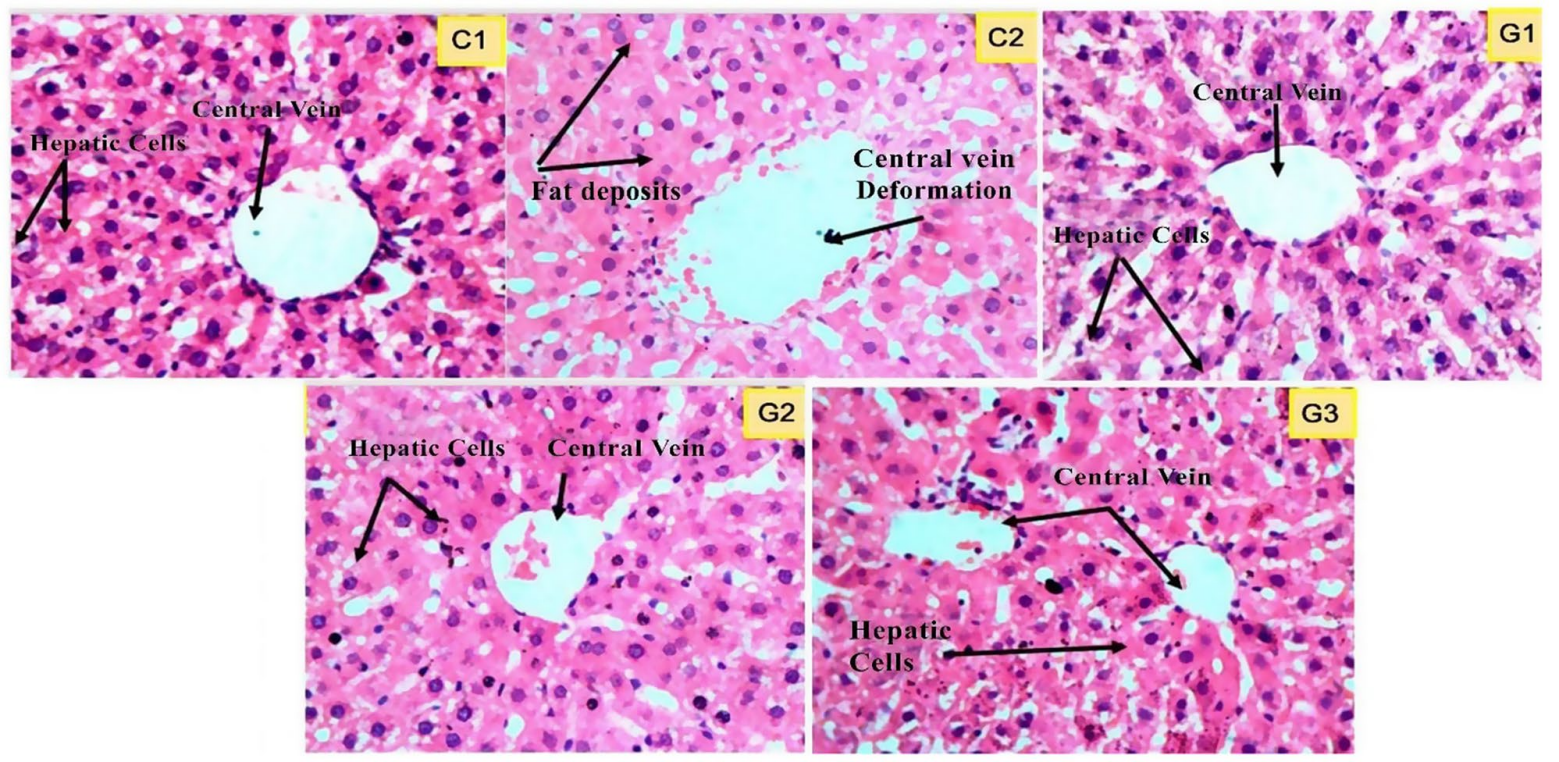
Photomicrographs of Hematoxylin and Eosin (H&E) stained sections of Liver of Probiotic and Fenofibrate fed rats (magnification 400X). Where, ^C1^Control, ^C2^HCD fed rats, ^G1^Probiotic Group, ^G2^Fenofibrate Group, ^G3^Probiotic + Fenofibrate Group

Histological examination (40 × 10X) of healthy liver tissues from the C1 group (control) revealed a typical portal vein size and hepatocyte architecture; no fat accumulation was observed in this group as well, and tissue structures were found highly organized in the histogram (Fig. 5). On the other hand, histological examination of the C2 revealed a grade 2 injury with scattered cellular contents due to disruption of the hepatocyte cell membrane, hepatocyte death, severe portal vein deformation, and heavy fat accumulation in the liver tissue. The G1 probiotic-treated group showed moderate restoration of hepatocellular structures, moderate portal vein size and less fibrous contents than C2. In contrast, liver tissues in the G2 (fenofibrate group) and G3 (Probiotic + fenofibrate Group) had evidence of a restored normal portal vein size and hepatocyte with well-organized architecture without any deposits of fat as compared to C2 as shown in the histogram.

## Discussion

CVD is the most common death cause worldwide. The Global Burden of Disease Study 2013 reported 15.6 million deaths worldwide, with CVD responsible for 30% [9]. The pathogenesis of CVD has been traditionally linked with high cholesterol levels starting from the time when the ‘lipid hypothesis’ was first presented [83, Zivkovic, 2023 #117]. Consequently, excess adipose tissue, non-alcoholic fatty liver disease (NAFLD), abnormal cholesterol levels, and impaired insulin sensitivity can increase susceptibility to cardiovascular disease [57]. CVD incidence has been on the rise in recent years in low-income countries like Pakistan, especially among the young, due to inadequate health and financial support [74]. Reducing cholesterol levels is crucial since even a minor decline (~ 1%) can help lower the risk of cardiovascular disease (CVD) by 2–3% [55]. Current common and widely accepted CVD treatment options include drugs and surgery, but both have limitations and downsides. Medication, though less invasive, can cause damage to vital organs such as the liver and kidneys as well as fatigue and muscle soreness [80]. In contrast, clinical procedures are limited by cumbersome procedures and the risk of postoperative infections [94].

In contrast, probiotics are an efficient, convenient, and cost-effective approach to treating CVD. Probiotic strains of *Enterococcus* have mainly originated from the gastrointestinal tracts of animals and humans, including their feces. Such enterococci are deemed more competitive than their counterparts from different settings, thus deserving of more significant consideration when prospecting for new probiotic candidates. A slew of enterococcal strains, including those of *E. durans*, are commercially available, claiming to provide multiple human health benefits. Yet the allure of isolating and characterizing novel strains remains strong because their benefits are highly strain-specific [61] [98]. *Enterococcus* strains remove cholesterol by incorporating it into the bacterial cell membranes during growth [80]. *E. durans*, in particular, is known to be a proficient cholesterol assimilator [14, 34, 49]. *E. durans* was also characterized for BSH enzyme function, which catalyzes bile acid deconjugation [70], facilitating their expulsion from the intestine through the feces [19, 31]. BSH activity significantly influences cholesterol absorption in the human intestine by deconjugating bile salts (Yerlikaya & Akbulut, 2020). Hydrolyzing bile salts can mitigate cholesterol micelles formation in the intestine, thus reducing serum cholesterol levels [1, 61]. Microbe-associated BSH activity affects the animal or human host’s circadian rhythms, its ability to gain weight, and prevents pathogens like *Clostridium difficile* from colonizing the gut [77].

BSH activity is a mode of defense that allows the bacteria to survive in the intestinal milieu. Reducing the toxicity of bile salts allows them to persist for extended periods [8, 32, 61]. In addition, amino acids released from bile salts could play a nutritional role, function as an energy source, and provide a shield against membrane-damaging compounds [69]. Recently, the role of BSH enzymes as amine N-acetyl transferases has been discovered. Production of bacterial bile acid amidates (BBAAs) in this way, whose functions remain largely unknown, is especially prominent in BSH-carrying *Enterococcus* sp [77]. Expectedly, the World Health Organization (WHO) has proposed that in addition to the conditions of being able to withstand harsh GIT conditions and colonize the intestinal epithelia, the presence of a functional BSH should be regarded as a critical factor in selecting probiotic candidates [79]. BSH-positive probiotics are increasingly considered candidates for controlling hypercholesterolemia by lowering circulating serum cholesterol levels [10]. The existence of *bsh* genes in the genome of *E. durans* is well known [17, 48]; also their role in deconjugating bile acids is documented [37, 97], but the magnitude of activity varies from strain to strain [61]. It has been argued that the ability of LAB spp. to lower gut pH might also facilitate BSH-mediated bile salt deconjugation and removal of luminal cholesterol [12]; however, the rate of cholesterol assimilation by LAB remains unaffected by variations in pH [66].

Most bacterial BSHs hydrolyze glycoconjugates of bile acids better than tauro ones [96], a feature that benefits the human host since taurine metabolism produces hydrogen sulfide, which is highly toxic [56]. A preference for deconjugating glycine-conjugated bile acids only, as is the case here, can be deemed desirable in a candidate probiotic strain [56]. This can also allow Tauro-conjugated bile acids, unaffected by microbial BSH action, to coax the sporulating bacteria to germinate, which helps improve dysbiosis caused by antibiotic exposure [11]. Varying substrate specificities of microbial BSHs have been linked to how vulnerable the microbial species is towards a particular bile acid. Thus, enterococcal strains or species, which are phenotypically insensitive to taurocholate, as is the case here, also lack BSH, which could hydrolyze them [76] Excess levels of secondary bile acids in the gut arising from transforming unconjugated primary bile acids via 7-α dihydroxylation by the gut microbiota is arguably a health concern. However, our strain of interest cannot contribute to this pool of secondary bile acids since the enterococci, like other LAB, cannot carry out this reaction [82] It is worth mentioning that secondary bile acids like lithocholic and deoxycholic acid are essential in preventing *C. difficile* proliferation and maintenance of immune and metabolic responses of the host [26] Novel BSH-producing probiotic strains are being increasingly sought out to identify agonists of FXR, a cell nucleus associated receptor involved in bile acid synthesis and transport [10].

Functional prowess notwithstanding, the would-be probiotic strains must be thoroughly assessed for their ability to survive and persist in the human GIT without causing harm to the host, which is the approach taken here [73]. This is usually accomplished by characterizing prospective strains using a battery of in vitro tests because of the many advantages they offer, yet they cannot reliably predict strain performance in vivo because of confounding animal and human GIT factors like gastric and intestinal juice release and composition, changes in pH, gut microbiota composition and frequency and intensity of peristalsis; therefore, in vivo assays are preferred since they can predict more accurately a would be probiotic strains nature [22]. An ability to colonize the GIT tract ensures that the exogenously delivered strains are not flushed out from the colon due to peristaltic movements [58]. Safety status is of paramount importance since the duality of the enterococcal nature makes it controversial [98].

Using rats for in vivo trials is advantageous because they are practical models for research on monogenic traits and diseases, as well as complex ones like cardiovascular diseases. Because of their larger size and well-characterized physiologies, rats have a distinct advantage when performing surgery and monitoring health status [84]. It is pertinent to mention that no universal protocol for inducing hypercholesterolemia in lab animals for CVD research has been put forth. However, the most typical approach recommends adding 1% cholesterol to the animal diet [18], which was also done here. Another intriguing advantage of using rats is that the elevation in serum levels of TC and TG following the provision of HFD to rats happens quickly in a few weeks instead of months [57] which we also observed here.

Probiotic efficacy is influenced by various factors, such as the type of strain used, disease severity, treatment duration and dose given. The dose of the test probiotic used in this study falls midway in the range deemed therapeutically effective, i.e., 10^8^-10^10^ cfu/day. Prominent food regulators like the Canadian Food Inspection Agency advocate using a dose of 10^9^ cfu/day of a single strain [36]. Half dose of fenofibrate was used in experimental G3 along with *E. durans* to study their combine effect. The moto behind was to explore that if fenofibrate amount was reduced to half and probiotic was given alongside can they provide the same effect as fenofibrate in its full potential. Oral gavaging of *E. durans* suspensions can ameliorate high cholesterol levels, both with and without fenofibrate in hypercholesterolemic rats as corroborated by past studies [30, 86, 91]. Reducing hepatic cholesterol and triglyceride levels can lower the conversion of intermediate-density lipoproteins to low-density lipoproteins, thus reducing serum LDL-C concentration [21]. HDL cholesterol levels were equivalent across all experimental groups but were considerably more significant than in the hypercholesterolemic group. Increased serum HDL levels following probiotic administration are known [16] Cholesterol addition to the diet tends to increase the body weight of experimental rats, as observed elsewhere [3]. The rat’s age is another factor affecting diet consumption and helps the animal gain weight [18]. However, the weight gain stopped following administration of probiotics and anti-hypercholesterolemic drugs, which aligns with past findings showing a drop in body weight of hypercholesterolemic rats following probiotic administration [42, 71, 93]. ALT and AST are clinical biomarkers that can be used to ascertain the level of liver damage [51]. Elevated ALT and AST levels, as seen here in rats given HFD, have also been linked to hepatic damage in rats. High levels of both indicate NAFLD and metabolic dysfunction like insulin resistance [39]. The occurrence of normal levels of ALT and AST in the treated groups, which is significantly less than that of the hypercholesterolemic group, highlights their safeness for use and lends credence to previous studies [41, 50, 63].

Hydroxyl (OH^−^) ions and other radicals are deemed harmful reactive oxygen species (ROS) produced by the Fenton reaction in the company of transition metals and cause oxidative injury to biological macromolecules. Excessive amounts of ROS impair cellular function and predisposes individuals to certain diseases [20]. Hypercholesterolemia causes alterations in cholesterol and triglyceride metabolism, weakening enzymes like catalase and superoxide dismutase, creating more ROS, which leads to more lipid peroxidation. The role of ROS in aggravating the pathogenesis of cardiovascular disease has been well characterized [18, 68]. Because of the inherent fickleness of the radical scavenging phenomenon, it is usually recommended that it is assessed using more than one approach [8], which is why multiple tests like ABTS, TAC, TOS and MDA were performed here. In a healthy individual, oxidative stress is typically handled by an arsenal of antioxidants generated by the body, supplemented by antioxidants gained from ingested food. However, in diseases like those described above, this natural antioxidant shield breaks down and must be restored by various means, including administering exogenous probiotics [2]. Antioxidant potential must be assessed at the strain level because this activity in probiotics is highly strain-dependent, and both the cell and cell-free portion must be taken into consideration since it can manifest both in the cells as well as their cell-free culture supernatants [2]. Metabolites generated by probiotics, the so-called postbiotics, are helpful in situations where administering live cultures is not feasible, as is the case with immuno-compromised individuals and those with impaired gut barriers [29]. It is worth mentioning that the antioxidative effect of probiotic supplementation can also be indirect, a consequence of their role in rebuilding microbial communities ravaged by disease [24, 60] of the finding here of a specific enterococcal strain capable of reducing TOS and TAC levels in the human host has a precedent where exogenously administered *E. durans* strains exhibit significant antioxidant activity [7, 17].

The GIT tract is where the actions of dietary components, including exogenously delivered probiotics, first manifest [67]. The small intestine is the bastion of LAB spp, including autochthonous enterococci, where they carry out deconjugation of glycine and taurine-conjugated bile acids [82]. Disruption of gut microbiota can contribute to and lead to the development of metabolic disorders. Getting a handle on changes in gut microbiota related to CVD complications can help develop strategies for reducing serum cholesterol levels and systemic inflammation [52] The gut microbiota is a crucial player in the metabolism of orally ingested drugs. They can impact the efficacy of cholesterol-lowering drugs by changing their bioactivity and concentration [89]. Because they influence lipid metabolism, the gut and fecal microbiota of the test rats were also analyzed. The administration of test probiotics also boosted the numbers of beneficial LAB spp. in the gut, alluding to their role in gut microbiota restoration, which was proposed elsewhere [2]. *Enterococcus duran’s* role in maintaining gut homeostasis has been suggested in the past [27], and our findings align with those involving fatty diet-fed rat models [15, 54, 75, 90]. A recent animal-based study demonstrating the administered fenofibrate’s growth-promoting effect on several species of gut-associated beneficial bacteria, except the enterococci [52], leads us to believe that the abundance of LAB colonies recovered in G3 could be attributable to the doses of the enterococci they have received during the trial.

Fenofibrate is a safe and well-tolerated drug that can improve lipid abnormalities, systemic inflammation, and correct endothelial function and is often recommended for CVD prevention as a supplement therapy (Tarantino et al., 2018). The liver is the first organ in the human body to be exposed to substances absorbed from the gut. It is the leading site of TG, fatty acid and glucose synthesis and is the principal regulator of overall nutritional homeostasis [64] It detoxifies toxic molecules, creates bile acids, and regulates energy and lipid metabolism [47] HFD-induced hypercholesterolemia causes damage to the animal’s liver and increases its weight.

Liver damage is associated with high fatty acid matter in the diet [18]. As seen here, the increased TG and LDL-C levels in the HFD-fed group indicate progressive liver damage appearing externally in the form of an enlarged and discolored organ. Lipid accumulation is also evident in the HFD diet-fed group compared to the control and probiotic-administered groups. Prolonged intake of HFD in rodents and humans accelerates the onset of NAFLD, characterized by lipid storage in hepatocytes as lipid droplets [64] Probiotics can revamp liver function, slow the onset of NAFLD by regulating the gut microbiota and reduce endotoxemia by lowering serum lipopolysaccharides [12]. Our study is consistent with prior findings that probiotic therapy lowered hepatic fat deposition in HFD-fed rats [64]. The hepato-protective effect of this particular strain of *E. durans* is evident by the histomicrographic imagery of the livers of probiotic-fed rats showing a typical tissue architecture with undamaged hepatic parenchyma. At the same time, a high-cholesterol diet causes the onset of inflammation and hepatic steatosis in animal models [57].

## Conclusion

To conclude, desi chicken’s gut-derived *E. durans* strain has a significant cholesterol-lowering effect on high-fat diet-fed hypercholesterolemic rats. This strain also caused weight loss and contributed to a healthier intestinal and fecal microbial balance. The results also showed that *E. durans* has no adverse effects on the livers of the examined animals, underscoring its safety in vivo. The *E. durans* strain possesses therapeutic potential against diet-induced hypercholesterolemia and weight management. However, the mechanisms responsible are complex, multifactorial and need further research.

## Data Availability

The DNA sequence data which supports the findings of this study have been deposited in NCBI-Gen Bank available at https://www.ncbi.nlm.nih.gov/ with accession numbers PP338721, PP338722, PP338723, PP338724, PP338725, PP338726, PP338727, PP338728, PP338729, PP338730, PP338731, PP338732, PP338733, PP338734, PP338735, PP338736, PP338737, PP338738, PP338739, PP338740, PP338741, PP338742, PP338743, PP338744, PP338745, PP338746, PP338747, PP338748, PP338749, PP338750, PP338751, PP338752, PP338753, PP338754.
